# Floods in Southeast Asia: A health priority

**DOI:** 10.7189/jogh.02.020304

**Published:** 2012-12

**Authors:** Jacqueline Torti

**Affiliations:** School of Public Health, University of Alberta, Edmonton, Alberta, Canada

Out of all the natural disasters, floods are the most common in both developed and developing countries, accounting for approximately 40% of all natural disasters [1,2]. Flooding has severe implications on human health before, during, and after the onset of a flood. Southeast Asia is a region that is especially prone to frequent and severe natural disasters [3]. The Association of Southeast Asian Nations is comprised of Cambodia, Laos, Thailand, Vietnam, Brunei, Malaysia, Indonesia, the Philippines, Singapore and Myanmar [4]. In this manuscript, I discuss why flooding is a problem is Southeast Asia and why I feel flooding warrants attention compared to other problems in the area due to the serious health impactions that arise as a result of flooding. I also explore why flooding warrants attention compared to other health concerns in the region.

## IMPORTANCE OF THE PROBLEM

The frequency and severity of flooding in Southeast Asia has increased over the past several decades. Flooding is a prominent issue that is currently affecting many regions in Southeast Asia, in particular Cambodia, Thailand, Vietnam, Laos, the Philippines, and areas surrounding the Mekong River. According to the United Nations Office for the Coordination of Humanitarian Affairs [5] it is estimated that 9.6 million people are currently affected by the flooding in Southeast Asia, with 5.3 million in Thailand alone. According to the National Committee for Disaster Management and the Department of Hydrology, the floods in Thailand are so severe that they have been labeled the worst floods in over 60 years [5]. Flooding in Southeast Asia raises many concerns for the health and well-being of those affected.

## MECHANISMS OF THE IMPACT OF FLOODING ON HUMAN HEALTH

The consequences of flooding on human health can occur before, during and after a flooding event. However, not many think about the health risks that can occur before a flood. There is an increased rate of mild injuries before the onset of a flood when people are advised to move their families to a safe location [3]. If left untreated, these injuries can have much larger implications on morbidity and mortality in the event of an actual flood, if an open wound is exposed to a source of contaminated water.

During the actual onset of the flood there is potential for direct causes of injury and death. Most deaths directly related to the onset of a flood are due to drowning, which frequently occurs as individuals attempt to move through high, fast moving water in their vehicles [4] or are swept away into moving waters while trying to help themselves of others [6]. Other deaths are the result of being struck by objects in fast moving water, dying of heart attacks, electrocution or by being buried in mud or collapsed infrastructure [6]. In 2011, an estimated 567 deaths due to floods occurred in Thailand, another 248 deaths in Cambodia, 85 deaths in Vietnam, 30 deaths in Laos, and 102 deaths in the Philippines with a total of 1, 302 deaths in Southeast Asia alone due to direct causes [5].

After the onset of a flood, there is potential for an increase in communicable diseases and further injury. As people start to accumulate back to their homes, the structure and foundation of buildings become damaged and weak, and collapsing can again cause injuries. Electric cables and wires may also be exposed or submerged in water increasing the risk of electrocution and burns [3,4,6]. Increased rates of carbon monoxide poisoning have also been reported as electric generators and pressure washers are used in the cleanup process. Many of these generators are fueled by petroleum and are used indoors or areas of poor ventilation [4]. There are also increased cases of hypothermia as individuals are continually exposed to frigid water, with parts of their body remaining wet for long periods of time [4].

The risk of communicable diseases, particularly fecal oral diseases, also increases in flooded areas. [3,6]. These diseases are spread when fecal matter is passed through the mouth and are more common in areas struck by floods due to declines in sanitation, lack of access to safe drinking water, and the consumption of contaminated foods. Other fecal–oral diseases that are of increased prevalence during a flood include typhoid fever, paratyphoid, poliomyelitis, hepatitis A and hepatitis E [3,4,6].

Vector–borne diseases also increase in prevalence after the onset of a flood, primarily with mosquitoes being attracted to stagnant and slow moving water for breeding purposes. [3,4,6]. Outbreaks of rodent–borne communicable diseases are also reported during floods and are mainly due to increased amounts of rodent excrement in flood water [3,4,7].

Mental health issues have also been known to increase in populations who have experienced flooding, most commonly anxiety, depression and stress [3,6]. I strongly feel that mental health issues are not given as much attention as they deserve. For many, the emotional trauma of experiencing a flood is overwhelming. Thousands of individuals have to relocate, often residing in government shelters [5] leading to a diminished sense of place and attachment, along with a loss of self–identity [4]. Posttraumatic stress disorder, suicide, sleeplessness, irritability, anger and schizophrenia are also common mental health issues that increase in burden during floods [3,6].

Malnutrition is another threat in flooded areas. Food security has become a major issue in Southeast Asia, as it has been difficult to deliver food supplies to flood–affected [8]. Severe damage to agricultural land and livestock affects those who rely on these forms of food production, with rice, maize and coffee beans severely damaged, along with losses in livestock and poultry [9]. This puts many people out of work, placing a financial strain on the economy and on many businesses and families [8]. Severe damage is also suffered by many health care centers, making access to medical care more challenging in the areas that need it the most [6].

## CAUSES OF THE PROBLEM

The recent floods in Southeast Asia are a result of a multitude of factors, including typhoons, heavy rains, and tropical storms [5,8]. The combination of four tropical storms and heavy and prolonged monsoon rains caused severe damage in Thailand, Cambodia, the Philippines, Vietnam, Laos, and Burma situated around the Mekong River [10,11]. The Asian monsoon is a wind system that changes directions with the seasons, and carries with it heavy rainfall [12].

Flash floods are a type of flood that occurs suddenly and progress just as quickly as it regresses; they are often short in duration, but their sudden onset makes them extremely difficult to predict and prepare for [13]. Water levels in both the Tonle Sap River and the Mekong River have been rising since August 2011. The overflow of these rivers, along with the combination of monsoon rains and tropical storms caused severe flooding in Southwest and Northern Cambodia, affecting 18 out of the 24 provinces [10].

On a larger scale, these heavy monsoon rains, typhoons and tropical storms are a result of climate change, a very complex system that is characterized by the dynamic relationships between land, bodies of water and living beings [14,15]. Southeast Asia is particularly vulnerable to the impacts of climate change due to its fast growing population, the majority of which are living in poverty, as well as poor food security and dwindling natural resources [14]. Climate change is a result of increasing temperatures which is linked to more intense rains, heat waves, extreme weather events, greater climate variability and rising sea levels, all which contribute to the increased frequency and intensity of flooding [14,16].

Global warming undeniably exacerbates flooding, however the extent of damage done by these floods along with their vast negative health implications are essentially due to particular issues in Southeast Asia. In some parts of South–East Asia there are problems with solid waste management systems. This can contribute to flooding in urbanized areas of Southeast Asia and can contribute to the spread of communicable diseases [17]. Limited resources within low–income countries make it difficult to prepare for flooding, as the resources are often not there to build sea defense, or other counter measures in areas that are at risk of flooding caused by rising sea–levels [18].

Many low–income countries in Southeast Asia are at a greater risk of poor health because a large number of people are living in crowded, highly urbanized areas, where they are more susceptible to contracting and spreading infectious diseases [18]. Disease control programs have improved over the years, however compared to high–income countries there is limited capacity to implement these programs effectively, thereby limiting low–income countries’ ability to deal with flood induced increases of these diseases [18].

## INTERVENTIONS TO ADDRESS THE PROBLEM

A variety of interventions have been implemented in response to recent flooding in Southeast Asia; some more successful than others. One of the key elements to flood intervention is prevention. In Bangkok the government had set up a sandbag wall that spanned over six kilometers in order to prevent flooding from high tides in November of 2011 [10].

Detection and early warning system technologies also play an important role in dealing with floods. The Pacific Disaster Center has designed a specialized early warning technology program for Vietnam called VinAWARE [5]. In 2011 alone, VinAWARE was able to detect and track the tropical storms “Nock–Ten” and “Neast” along with several other tropical depressions. It also led to the production of over 950 flood advisories, over 250 storm advisories and over 2200 strong wind advisories, potentially avoiding disastrous results [5].

Detecting and tracking these natural disasters is only the first step. The next important steps involve awareness and community preparedness. Both governmental and nongovernmental organizations in Asia have engaged in small–scale mitigation. However, these efforts are often unsustainable due to a lack of connectedness and engagement with the community [19]. Other challenges to sustaining community preparedness through these organizations include a lack of funding and donor exhaustion. With limited resources, concrete action is often neglected while training and planning takes precedence [19]. There is a strong need to allow communities to take action themselves. For example, the Intermediate Technology Development Group–Bangladesh [ITDG–B] has created an improved housing model and has worked with community members to raise the foundations of homes, treat building materials in order to promote durability and to ensure houses being built have windows for proper ventilation [19,20]. Another community preparedness method is modifying agricultural practices to prepare for floods. Examples include seed preservation and the planting of flood resistant trees in order to protect agricultural production and improve food security during and after the time of a flood [20]. The health and well-being of livestock during a flood is also an important consideration. Southeast Asian communities are designating space for livestock in flood shelters, preparing and storing emergency feed, as well as de–worming and vaccinating farm animals [15,19]. More refugee sites are also being developed along with an evacuation plan, however, negotiations with local union parishad bodies and governmental officials were unsuccessful [20].

Community preparedness plays a strong role in assisting with emergency aid during the onset of a flood. One of the first emergency responses during a flood is evacuation. Evacuation procedures have been set in place, as part of community preparedness, to move people away from flooded, at risk, or dangerous areas. Many roads in Asia are raised to a level that is higher than annual floodwaters allowing people and livestock to travel safely to high grounds or shelters. In fact, in Vietnam, the government has created a successful program known as *flood kindergartens*, which provide parents or guardians with a safe place to leave their children when flood warnings are administered [21]. However, evacuation efforts can be exhausted as many people neglect to evacuate because they fear losing their home and their belongings. Therefore, asset protection is another major feature of emergency response. Many agencies in Asia practice asset protection including such things as storing feed for livestock, replacing lost livestock, rebuilding communities and houses, and distributing tools needed to maintain and reconstruct farms and other businesses [19]. Other solutions include having the police or military secure flooded areas until it is safe for people to return to their homes.

Other forms of emergency response include search and rescue expeditions. These expeditions are extremely complex and need to be carried out in a very efficient and timely manner. The Sphere Project’s Humanitarian Charter and Minimum Standards in Disaster Response and the United Nations High Commissioner for Refugees [UNHCR] plays a key role and search and rescue, setting standards for care and operational guidelines for triage units [22].

Other health concerns besides emergency first aid also need to be addressed. These include issues of communicable disease transmission, sanitation, food security and access to safe drinking water. The World Health Organization’s Southeast Asia office has reported the need to improve the monitoring and surveillance of disease outbreak during floods [23]. Various initiatives have been set in place to improve sanitation during floods including the provision of safe sites for human excretion, promoting awareness, educating the community on safe sanitation practices and providing soap for hand washing. However, I feel food security in Southeast Asia is still an issue that needs improvement. The Agreement on Disaster and Emergency Response and the Coordinating Centre for Humanitarian Assistance on Disaster Management are two mechanisms set in place to address the need for improved food security [24]. On October 7, 2011 the Association of Southeast Asian Nations collaborated with Northeast Asia to create the ASEAN Plus Three Emergency Rice Reserve, in which 787 000 tons of rice was preserved for emergency use [24]. This collaboration along with the assistance of the UN World Food Program [UNWFP] is likely to have a positive impact on food security in Southeast Asia [23]. Improving sanitation and reducing risk after the onset of a flood are also important measures that need to be taken in order to minimize negative health impacts. For example, the government in Thailand is working alongside the Bangkok Metropolitan Administration to drain floodwaters from Bangkok rivers into the Gulf of Thailand [5]. This will decrease water levels, decrease the risk of transmitting communicable diseases, and help to protect assets of livelihoods of the people residing in central Bangkok.

Operational assistance and monetary donations assist in dealing with negative implications of flooding. The Emergency Operations Centre plays a strong role in assisting with emergency response by providing direction and strategic management [21]. The United Nations and government agencies also play their part by providing funding, resources and support for disaster management [21]. In November of 2011 the UNWFP provided funding of over US$ 490 000 to provide operational assistance for emergency response in Southeast Asia [10]. The United Nations allocated US$ 4 million to Cambodia towards the United Nation Central Emergency Response Fund [10]. These projects have been successful in Southeast Asia because of coordination efforts at both the national and local level [19].

Operational or monetary assistance are also being used to examine the existing needs in areas affected by flooding. For example, in Vietnam, the United Nations, governmental and nongovernmental organizations are working together to establish any gaps or remaining needs for recovery [5]. Another example is in Thailand where the United States Agency for International Development along with the Office of Foreign Disaster Assistance are conducting research to examine remaining needs in affected areas [10].

Many interventions have been set in place to address the implications of flooding in Southeast Asia. Some interventions have been highly successful due to collaboration, coordination, and community engagement. However I feel some interventions have failed to address all the needs of this population and require further attention, action and improvement.

## POLICY RECOMMENDATIONS

### Community preparedness

Community preparedness is an example of an intervention that can be improved in Southeast Asia. Both governmental and nongovernmental operations are often unsustainable due to both lack of funding and community engagement [19]. In order to improve community preparedness relationships between these organizations and the community should be improved and maintained through ongoing support and guidance [19]. There is a strong need for governmental organizations to continue to develop flood preparation strategies beyond mitigation and adaptation to include such measures as monitoring, warning, communication and dissemination, and planned evacuations [25].

### Risk assessment

I think improvements also need to be made in the area of risk assessment. The concept of risk assessment needs to be changed to include such measures as adaptation strategies and to recognize not only the role of geography, or location, but to also recognize socially constructed vulnerability [25]. Along with risk assessment there is also the issue of risk reduction, which is often limited in terms of funding and resources, but it is important to achieve it through awareness, education, dissemination, and community preparedness and to keep it in mind during health system planning [6]. Risk reduction issues are often neglected in comparison to emergency response. Health systems need to learn from the hazards involved in past flood experiences in Southeast Asia and adapt to take precautionary measures before the onset of a flood. To date, there is little evidence of this process [6]. Although knowledge translation can be context–specific, there is a need to learn from past experiences around the world and ensure risk response and adaptation strategies are disseminated and communicated about on a global scale [6].

### Communication and coordination

Communication and coordination are important factors that need to be considered when addressing floods and other natural disasters. In terms of risk assessment, risk reduction, community preparedness and risk response there is a strong need to improve communication and coordination between policy makers, governmental and nongovernmental agencies, donors, and local communities [6,19]. Information on flood risks needs to be disseminated in a lay and culturally appropriate to reach community members in an effective way. There also needs to be an increase in community input when making decisions and planning emergency response strategies [6]. Governmental and nongovernmental organizations also need to develop stronger coordination and communication when assisting these communities in order to avoid duplication and ensure that the areas that are most affected are receiving the assistance they need [19].

There is also room for improved communication and coordination when assessing the implications of flooding on a population. Organizations tend to have their own research agendas and often neglect to coordinate with other organizations when performing assessments, making it difficult to generalize results. This leads to repetition and replication causing greater post–traumatic stress to flood victims as they are being asked to recall their experience on multiple occasions, which becomes emotionally exhausting [21]. There is also a need to incorporate flooding when formally assessing health implications of climate change.

### Global action

Given the extensive damage flooding has caused in Southeast Asia, I think it is imperative to learn from these experiences and promote global action. However, implementing best practice on a global scale requires extensive political will and monetary resources, which are not always readily available [6]. For example, limited funding is available for research being done by nongovernmental organizations in the Philippines, making capacity building and community preparedness strategies difficult to implement [6]. The majority of resources are put into disaster relief operations while risk reduction and community preparedness are neglected. Nonetheless, it is essential that community preparedness and risk reduction strategies be granted attention, as they are typically low–cost efforts that can be implemented at the local and community level. However, this is a difficult ideological transition for many policy–makers and donors [6].

Other changes required on a global scale involve improving health infrastructure, including the quality and effectiveness of services being offered. In order to reduce health risks from flooding, health systems need to be strengthened, along with other life–supporting services [6]. During a flood, providing effective health care services can be challenging because the flood may also directly affect both staff and facilities. However, there is a need to improve awareness of exacerbated health impacts that arise with flooding and a need to improve continued care, long after a flood has occurred [4]. Improvements also need to be made in infectious disease control, sanitation, and food security to reduce hazardous outcomes during a flood. By improving the general health of a population, people become less vulnerable to health risks during the onset of a flood [6].

**Figure:**
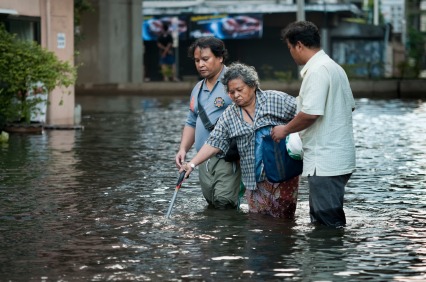
Photo: © istockphoto.com/ Giedrius Dagys

### Research gaps

There are many gaps in the research that has been conducted on flooding, including gaps in health risk reduction knowledge, as well as the gaps in understanding how we can reduce the implications of climate change, which plays a significant role in the presence of natural disasters, especially floods. In terms of health risk reduction during floods, further research needs to be conducted to determine the mental health implications of flooding. Little research has been done to examine long–term health implications, even in high–income countries [3]. This research would be important in order to learn how to provide counseling and continued care long after a flood has occurred. Mortality risks and risks of infectious disease also require research, as there is hardly any quantifiable research in this area [3].

Further research is also needed to determine to what extent climate change adds to flood risks and related health risks in various settings [3,26].

### Conclusion

Poor support and emergency services, along with a lack of resources in Southeast Asia largely contribute to negative health impacts of flooding in the area. On a larger scale, climate change may be increasing the frequency and severity of floods experienced around the globe. Many interventions have been set in place to prepare communities for floods, reduce and assess risk, and perform emergency services and aid. However, many of these interventions lack sustainability and the resources needed to provide lasting and effective services to those in need. I think the study of health implications of flooding and climate change are a relatively juvenile and there are many knowledge gaps in the literature that need to be addressed through further research and exploration.
